# High Stability and Long Cycle Life of Rechargeable Sodium-Ion Battery Using Manganese Oxide Cathode: A Combined Density Functional Theory (DFT) and Experimental Study

**DOI:** 10.1021/acsami.0c21081

**Published:** 2021-02-25

**Authors:** Bidhan Pandit, Sachin R. Rondiya, Nelson Y. Dzade, Shoyebmohamad F. Shaikh, Nitish Kumar, Emad S. Goda, Abdullah A. Al-Kahtani, Rajaram S. Mane, Sanjay Mathur, Rahul R. Salunkhe

**Affiliations:** † Institut Charles Gerhardt Montpellier (ICGM), Université de Montpellier, Place Eugène Bataillon, Montpellier 34095, Cedex 5, France; ‡ School of Chemistry, Cardiff University, Main Building, Park Place, Cardiff, CF10 3AT, Wales, United Kingdom; § Department of Chemistry, College of Science, King Saud University, P.O. Box 2455, Riyadh 11451, Saudi Arabia; ∥ Department of Physics, Indian Institute of Technology Jammu Jagti, P.O. Nagrota, NH 44, Jammu 181221, J & K, India; ⊥ Fire Protection Laboratory, National Institute of Standards, 136, Giza 12211, Egypt; # Swami Ramanand Teerth Marathwada University, Nanded, 431606, M.S., India; ∇ Chemistry Department, Institute of Inorganic Chemistry, University of Cologne, Greinstr. 6, 50939, Cologne, Germany; ● Department of Materials Science and Engineering and Chemical Engineering, Universidad Carlos III de Madrid, Avda. Universidad 30, E-28911 Leganés, Madrid, Spain

**Keywords:** MnO_2_, Rietveld refinement, DFT analysis, sodium-ion battery, life-cycle performance

## Abstract

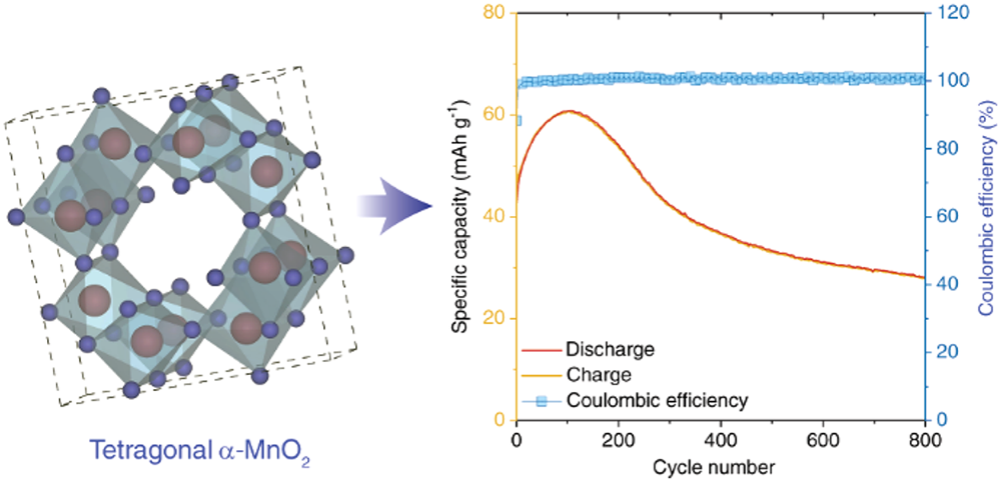

Sodium-ion batteries (SIBs) can develop cost-effective and safe energy storage technology for substantial energy storage demands. In this work, we have developed manganese oxide (α-MnO_2_) nanorods for SIB applications. The crystal structure, which is crucial for high-performance energy storage, is examined systematically for the metal oxide cathode. The intercalation of sodium into the α-MnO_2_ matrix was studied using the theoretical density functional theory (DFT) studies. The DFT studies predict Na ions’ facile diffusion kinetics through the MnO_2_ lattice with an attractively low diffusion barrier (0.21 eV). When employed as a cathode material for SIBs, MnO_2_ showed a moderate capacity (109 mAh·g^–1^ at C/20 current rate) and superior life cyclability (58.6% after 800 cycles) in NaPF_6_/EC+DMC (5% FEC) electrolyte. It shows a much higher capacity of 181 mAh·g^–1^ (C/20 current rate) in NaClO_4_/PC (5% FEC) electrolyte, though it suffers fast capacity fading (11.5% after 800 cycles). Our findings show that high crystallinity and hierarchical nanorod morphology of the MnO_2_ are responsible for better cycling performance in conjunction with fast and sustained charge-discharge behaviors.

## Introduction

The ever-increasing need for clean energy is driving the demand for large-scale energy storage devices made of earth-abundant, cost-effective, and environmentally benign materials. The lithium-ion battery (LIB) is the most perfected technology and a feasible option for short-to-medium scale energy solutions among existing battery technologies.^1−5^ It is useful in terms of high theoretical capacity (3860 mAh·g^–1^),^6^ cycle stability (∼30 000 charge-discharge cycles),^7^ and excellent rate capability.^8^ However, concerns about LIB safety, lifetime, and high cost limit its large-scale deployment.^9^ Although recycling of LIBs provides an attractive solution to reuse lithium, it is not a feasible solution considering the massive requirements for electric vehicles (EVs) and many other electric appliances.^10^ Thus, alternative battery technologies beyond LIBs is necessary to meet the goal of energy storage.

Sodium-ion batteries (SIBs) have emrged as promising alternatives for LIBs because sodium is widely available and exhibits similar properties as lithium.^11^ However, the larger ion size of sodium (Na, 1.02 Å compared to Li, 0.76 Å)^12^ and its higher ionization potential limit sodium for insertion into crystalline materials and energy density. Very recent investigations demonstrated that open structures could generally better accommodate the more abundant Na^+^ ion in terms of phase stability.^13,14^ However, suitable electrode material for SIBs remains elusive. For further advancement in Na-intercalation compounds, the development of novel materials by cost-effective and green synthesis routes is a practical solution. It is essential to mention that Na ions cannot easily intercalate into the graphite nanoarchitecture (while shown for other carbon nanostructures).^15^ This calls for developing new positive electrode (cathode) materials that largely determine the capacity, power density, and cyclability. An extensive search for new and advanced cathode materials has led to different crystalline materials and chemical compositions.

Among various transition metal oxides, manganese oxide has attracted significant attention because of the diversity of chemical compositions and structures existing in the Mn:O phase diagram. Also, the variable oxidation states of manganese can generate more than one electron in redox reactions. The other promising electrode properties include elemental abundance, low cost, easily tuned electrochemical properties, and low toxicity.^16^ Manganese oxides (MnO_2_) have large-sized channels that could facilitate Na ion intercalation.^17^ Other studies for MnO_2_ showed a capacity of ∼130 mAh·g^–1^ along with stable cycling performance.^18−20^ For instance, the first cycle capacity of 150 mAh·g^–1^ was reported for layered P2-Na_0.6_MnO_2_.^21^ Another study showed a reversible size of 156 mAh·g^–1^ with high Coulombic efficiency for Na_2/3_Fe_1/2_Mn_1/2_O_2_@graphene composites via a filtration process;^22^ however, these materials showed more than 50% capacity loss after only a few tens of cycles. In this context, manganese dioxide with different polymorphs and large open channels can accommodate guest cations.^23^ Up to now, the literature reports suggested that MnO_2_ could be a potential candidate for Na-ion batteries; however, cycling stability is a persisting issue for all of them.

Moreover, suitable electrolytes exhibiting good ionic conductivity, a sizable potential window, no reactivity toward the cell components, low toxicity, and a large thermal stability window are prerequisites for new cathode materials.^24,25^ These features depend on the solvent(s), nature of salt, and their additives. As for other technologies, SIB electrolytes require solvents with a wide range of liquid temperature, the high value of dielectric constant (*ε*_*r*_), low desolvation energy, low viscosity, chemical stability against electrode material, wide electrochemical window, and the ability to build a stable and suitable solid electrolyte interface (SEI).^26^ Cyclic and linear carbonates, mostly propylene carbonate (PC), ethylene carbonate (EC), and dimethyl carbonate (DMC), have been proposed as solvents for electrolytes.^27,28^ By comparison with lithium counterparts, NaClO_4_ and NaPF_6_ and more complex sodium salts were employed for this study. It is possible to avoid solvent molecules’ co-insertion by the stable surface film.^29^ As SEI film is useful to prevent co-insertion, its stability is critical for the cycle life of the material.^30^ Furthermore, to reduce the capacity loss and improve the cycle life, changing the surface composition by adding some film-forming additives is required. They are chosen specifically to create an SEI on the negative electrode, which passivates it and allows capacity retention upon cycling. For instance, Fluoro Ethylene Carbonate (FEC) has been a popular and most advantageous additive in SIBs as it could improve the efficiency and stability of half-cells.^31,32^ However, it introduces cell polarization.^27,33^ It is reported as a beneficial additive because of the formation of a more stable SEI film.^34^ Thus, additives play a vital role in improving SEI film stability.^35^

In the present study, the influence of anions on the performance of SIBs by comparing Na salts containing different counterions such as hexafluorophosphate (PF_6_^–^) and perchlorate (ClO_4_^–^) is studied. These two Na salts are common salts having the advantage of the differences in anion size (*PF*_6_^–^ = 0.51 nm, *ClO*_4_^–^ = 0.49 nm), comparatively stable anion structures, and ionic conductivities. First, the influence of electrolytes was carefully studied using a half-cell configuration (vs Na metal). Further, to evaluate the individual contributions of electrolytes to the individual electrodes, a comparative survey of electrolytes prepared using solvent (PC) and a solvent mixture (EC:DMC) combined with different Na salts, NaClO_4_, and NaPF_6_ was carried out. The electrolyte mainly controls the transport properties and adsorption behavior on the electrode surface. Thus, our results demonstrate that the electrolytes play a significant role in achieving high energy density along with good cycle life.

## Results and Discussion

### Crystal Structure and Morphology Analysis

**Figure 1:**
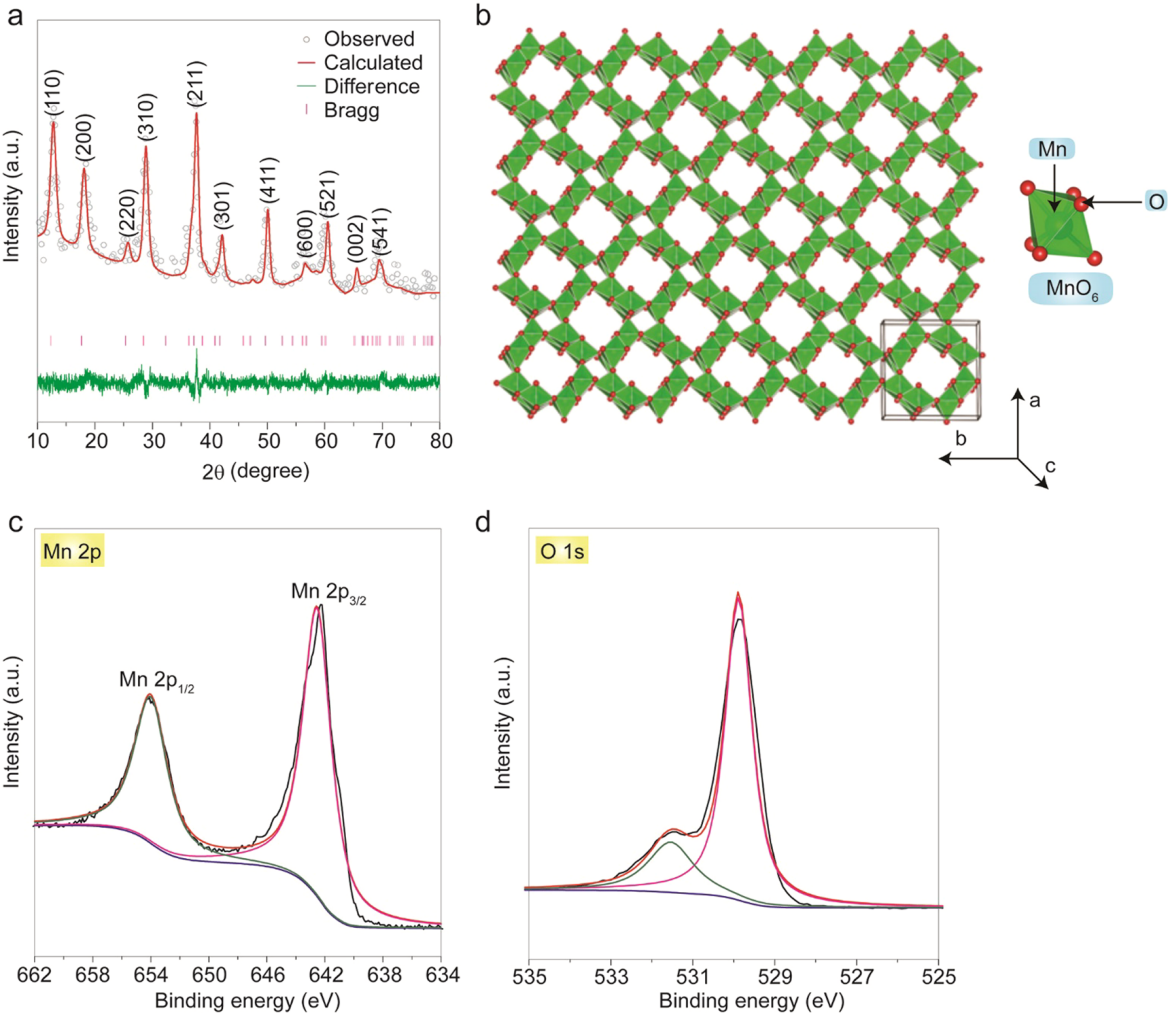
Structural analysis of MnO_2_ nanorods. (a) Rietveld refinement showing data points (light gray circles), calculated profile (red line), Bragg peak positions (pink vertical lines), and difference profile (green line), as indicated. Refined parameters are *a* = *b* = 9.815 Å, *c* = 2.847 Å, and α = β = γ = 90° with a unit cell volume of 274.264 Å^3^. (b) Crystal structure showing the species galleries for Na^+^ intercalation between MnO_2_ layers. A dark green color indicates the MnO_2_ layers. Small red spheres represent the oxygen atoms, and the Mn atoms in MnO_6_ polyhedra are depicted in green color. (c) Mn 2p and (d) O 1s XPS spectra of MnO_2_ sample.

The Raman spectrum (Figure S1) of the sample was measured in the frequency range of 100–1100 cm^–1^. The spectrum of α-MnO_2_ exhibited three prominent bands at 179, 583, and 641 cm^–1^. The Mn-O symmetric stretching vibrations of the [MnO_6_] octahedron leads to a peak around 641 cm^–1^. The peak 583 cm^–1^ is due to Mn-O stretching vibrations.^36^ The peak at 179 cm^–1^ is ascribed to external vibration of the [MnO_6_] octahedral.^37^Figure 1a shows the XRD patterns of the MnO_2_ nanorods with diffraction peaks at 12.90°, 18.21°, 25.76°, 28.84°, 37.72°, 42.15°, 50.04°, 56.20°, 60.20°, 65.50°, and 69.43° that correspond to (110), (200), (220), (310), (211), (301), (411), (600), (521), (002), and (541) crystal planes of α-MnO_2_, respectively (JCPDS: 44-0141). This validates the formation of phase pure tetragonal α-MnO_2_.^38^ The average crystallite size of the sample is determined to be 13.2 nm using the Debye–Scherrer method. The profile coefficients and Rietveld refinement of the lattice parameters show agreement between the experimental and a calculated model for the *I*4/*m* space group. The Rietveld refinement cell parameters resulted in *a* = *b* = 9.815 Å and *c* = 2.847 Å, leading to a cell volume of 274.264 Å^3^ for the material. The manganese oxide structure has 1D voids formed by the corner and edge-sharing MnO_6_ units adopting an *I*4/*m* tetragonal crystal structure (Figure 1b). Among various polymorphs of MnO_2_, the α-MnO_2_ tunnel structure is attractive for secondary battery cathode material application. This might be due to that the 1D structure combined with the high oxidation state of electroactive manganese provides an opportunity for (de)insertion of ions.^20,39^ The XPS analysis (Figure 1c,d) showed the spin-orbit doublet of Mn 2p centered at 642.5 (Mn 2p_3/2_) and 654.0 eV (Mn 2p_1/2_). The doublet peaks show a separation of 11.5 eV, indicating the existence of the Mn^4+^ oxidation state.^40−42^ The oxygen 1s spectra (Figure 1d) is deconvoluting into two, which indicate the presence of lattice oxygen (O_α_, 529.5 eV) and defect oxide or the surface oxygen ions (O_β,_ 531.5 eV).^43,44^ The details of XPS peak deconvolution are as per Note S1 in the Supporting Information.

**Figure 2:**
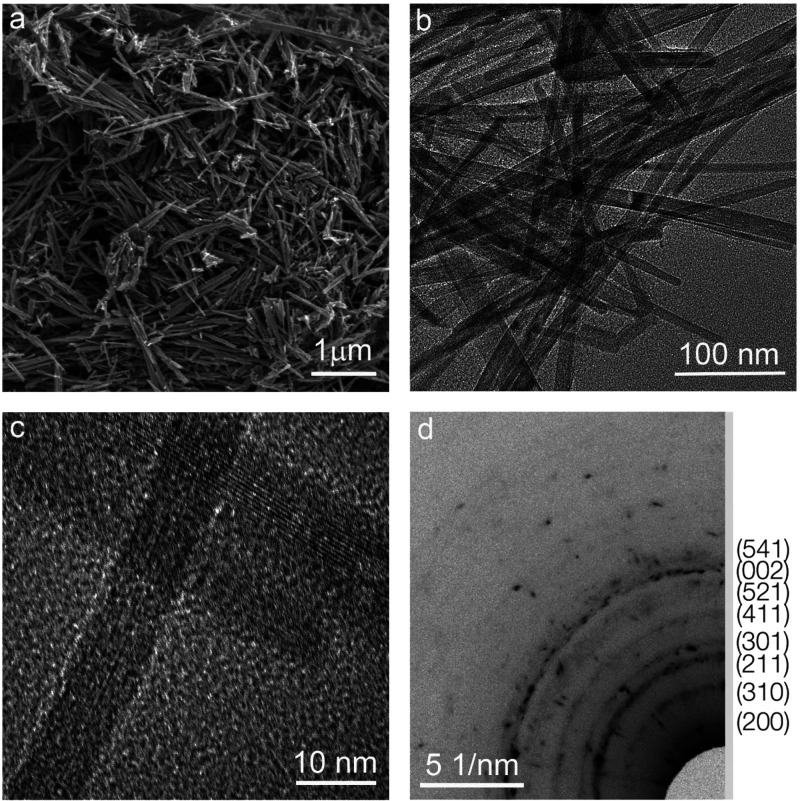
Electron microscopy analysis for the MnO_2_ nanorods. (a) Panoramic SEM image for the MnO_2_ sample showing their high degree of homogeneity in the sample. (b) TEM image of nanorods. (c) Lattice-resolved HRTEM image. (d) The bright-field SAED pattern of the MnO_2_ sample.

The FESEM image revealed the morphology of α-MnO_2_ shown in Figure 2a. It was observed that α-MnO_2_ consists of nanorods like one-dimensional architectures with an average diameter of ∼10 nm and an average length of 5–10 μm, as confirmed by the TEM analysis of the nanorod (Figure 2b,c). Besides, SAED patterns demonstrated the coexistence of fine particles and the crystalline nature of α-MnO_2_ nanorods (Figure 2d). The SAED patterns clearly showed the formation of rings, which can be indexed to the (200), (310), (211), (301), (411), (521), (002), and (541) planes of α-MnO_2_ (JCPDS: 44-0141). The purity of the samples has been investigated by EDS elemental mapping analysis (Figure S2a–c). The presence of Mn and O in their optimal stoichiometry indicates the formation of α-MnO_2_ nanorods without any impurities. These nanorods’ unique morphology shows the high surface area for electrolyte ions to penetrate, resulting in enhanced electrochemical performance.

### Electrochemical Performance Studies

**Figure 3:**
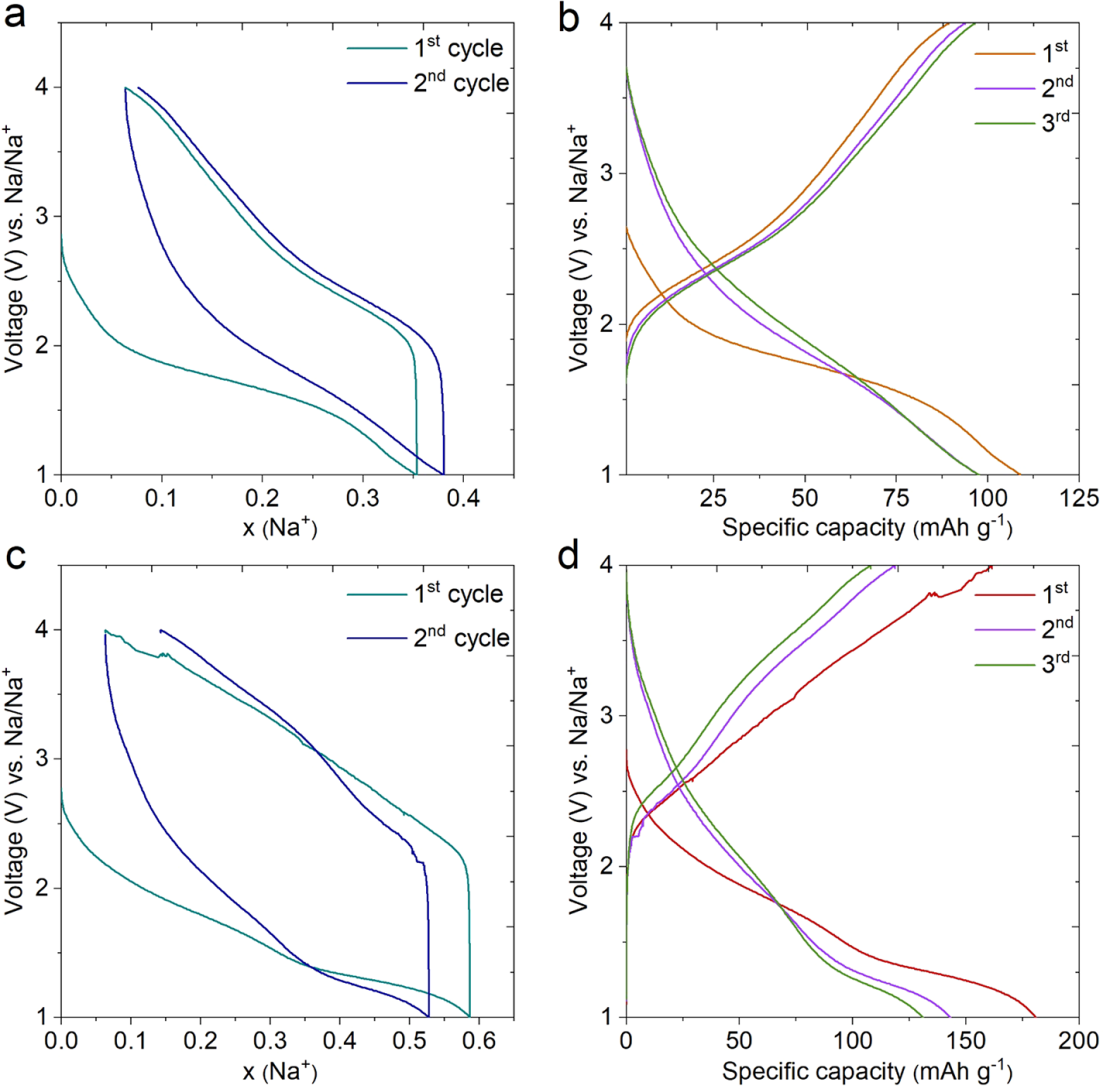
Electrochemical sodium storage capacity of MnO_2_ nanorods for half sodium cells. (a) Electrochemical charge-discharge curves per Na^+^ ion, and (b) GCD profiles of MnO_2_ at C/20 current rate in NaPF_6_ in EC:DMC in 5% FEC electrolyte. (c) Comparative charge-discharge curves per Na^+^ ion of the same material in NaClO_4_ in PC with 5% FEC electrolyte, and (d) GCD profiles of MnO_2_ at C/20 current rate in NaClO_4_ in PC with 5% FEC electrolyte.

Na^+^ insertion/extraction into the MnO_2_ matrix was evaluated by galvanostatic charge-discharge (GCD) tests within the potential of 1–4 V. To test the mechanism, two electrolyte systems, including NaPF_6_ in EC:DMC in 5% FEC and NaClO_4_ in PC with 5% FEC systems, were evaluated for the MnO_2_ electrodes; the results are shown in Figure 3. Figure 3a,c shows the first discharge curves of the MnO_2_ electrode at C/20 (C/20 = 1 Na in 20 h). The discharge curve shows a large voltage plateau up to 1 V, corresponding to Na ion insertion into the lattice vacancy of MnO_2_. As seen in Figure 3a, 0.35 sodium can be inserted into MnO_2_ in the case of using NaPF_6_ in EC:DMC in 5% FEC during the first discharge at C/20 rate between 4 and 1 V vs Na^+^/Na, out of which only 0.29 sodium can be removed on the following charge, leading to reversible operational capacity. In the case of NaClO_4_ in PC with 5% FEC electrolyte, there is 0.59 sodiation. An approximate irreversible capacity was 0.06 Na during the first charge for the cutoff voltage of 4 V. As shown in Figure 3b, for the initial discharge process, the MnO_2_ electrode achieved a high capacity of 109 mAh·g^–1^, with a charge capacity of ≈89.3 mAh·g^–1^ and Coulombic efficiency of 81.9%. The remaining discharge and charge capacities were 97.7 and 96.4 mAh·g^–1^, respectively, after the third cycle. In the case of the NaClO_4_/PC (5% FEC) electrolyte, MnO_2_ delivers a discharge and a charge capacity of 181 and 161.5 mAh·g^–1^, respectively, with a Coulombic efficiency of 89.2% (Figure 3d). After three cycles, the reserved discharge and charge capabilities were 131.1 and 108 mAh·g^–1^ with a polarization phenomenon, as observed in the previous one. The improved faradaic kinetics leads to achieving better capacity.

The open channels of MnO_2_ enable decreased ion diffusion paths and thereby fast electron transfer. The specific capacity observed for the MnO_2_ electrode in a half-cell is high in the NaClO_4_/PC electrolyte system compared to NaPF_6_/EC+DEC, and it decreases in the following cycles, although the Coulombic efficiency is maintained at 100% in both electrolytes. Given its excellent electrochemical properties, PC appears to be a promising nonaqueous electrolyte candidate for use in stationary SIBs.^45^

**Figure 4:**
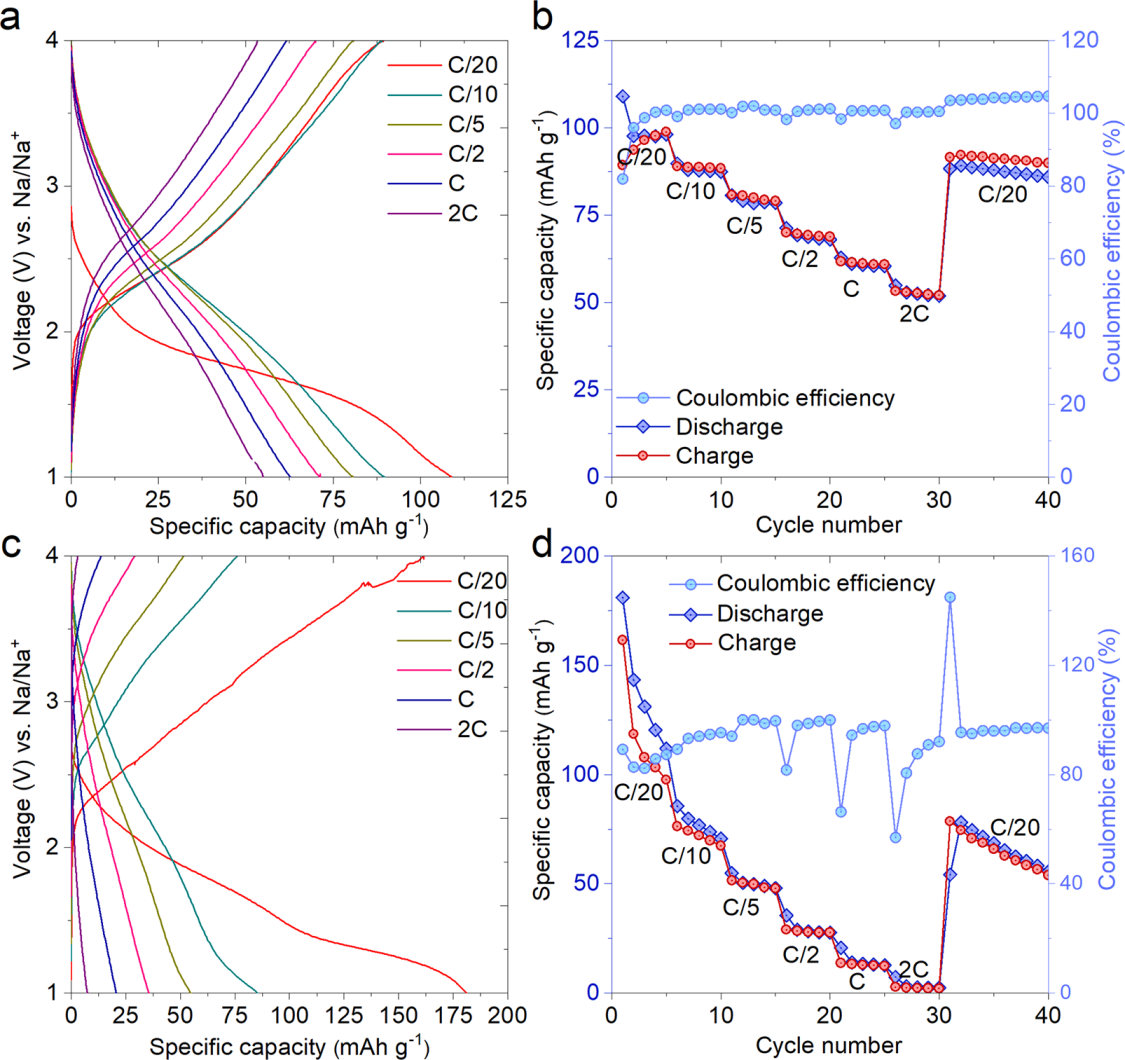
Comparative studies for different electrolytes. (a) GCD profiles of MnO_2_ cathode at different C-rates, for C/20 to 2C rates in NaPF_6_ in EC:DMC in 5% FEC, and (b) rate capability at varying C-rates and corresponding Coulombic efficiencies in NaPF_6_ in EC:DMC in 5% FEC. Furthermore, studies have been carried out for (c) GCD profiles of MnO_2_ cathode at different C-rates, for C/20 to 2C rates in NaClO_4_ in PC with 5% FEC electrolyte, and (d) rate capability at varying C-rates and corresponding Coulombic efficiencies in NaClO_4_ in PC with 5% FEC electrolyte.

Further, rate performance studies for the MnO_2_ sample were carried out stepwise from C/20 to 2C. The observed capacities are 109, 89.7, 80.6, 71.2, 62.8, and 54.9 mAh·g^–1^ at current rates of C/20, C/10, C/5, C/2, C, and 2C, respectively, for NaPF_6_ electrolyte (Figure 4a). It is worth noting (Figure 4b) that the specific capacity nearly recovered the initial value of 88.3 mAh·g^–1^ along with the Coulombic efficiency of 100% at the reverse current rate of C/20, indicating the excellent structural stability and reversibility of the MnO_2_ sample. MnO_2_ showed a discharge capacity of 181, 85.5, 54.8, 35.6, 20.7, and 7.3 mAh·g^–1^ at current rates of C/20, C/10, C/5, C/2, C, and 2C, respectively, in NaClO_4_ electrolyte, which is much better as compared to the results in NaPF_6_ electrolyte (Figure 4c). The high capacitance of the MnO_2_ electrode in PC compared to EC+DMC is derived from ion diffusivity, improved conductivity, and porous conductive matrix engagement. There is no apparent capacity fading tendency for the case of NaPF_6_ in EC+DMC electrolyte; however, MnO_2_ displayed severe capacity degeneration in NaClO_4_ in PC as shown in rate capability studies (Figure 4d). MnO_2_ exhibited poor cycle stability at the high current density and stable performance at low current density.

**Figure 5:**
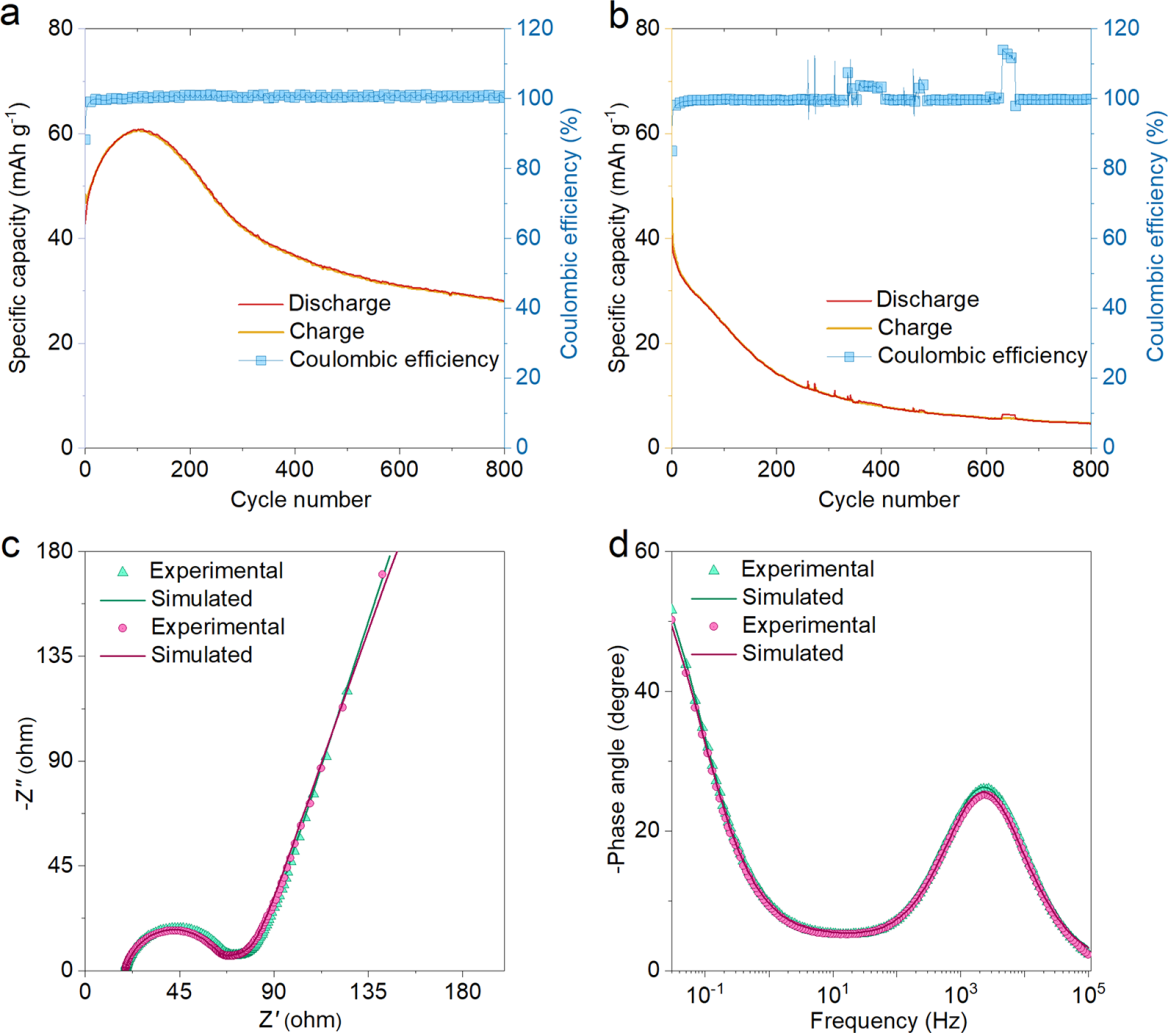
Extended cycling performance studies. (a) Extended cycling performance and the corresponding Coulombic efficiency at 1C-rate showing ∼58.6% of capacity retention and is maintained after 800 cycles in NaPF_6_ in EC:DMC in 5% FEC. (b) The comparative stability test of the same sample in NaClO_4_ in PC with 5% FEC electrolyte shows that capacity decreases abruptly after 200 cycles; this is a consequence of large polarization on the MnO_2_ surface due to the decomposition of PC during the charging process. (c) The Nyquist plots and (d) Bode plots for corresponding NaPF_6_ in EC:DMC in 5% FEC (violet in color) and NaClO_4_ in PC with 5% FEC (green in color) electrolytes.

The extended cycling tests of the MnO_2_ electrodes in the NaPF_6_/EC+DMC and NaClO_4_/PC electrolytes are as shown in Figure 5a,b. The capacity retention of 58.6% after 800 cycles can be observed in NaPF_6_/EC+DMC electrolyte at the current rate of 1C, whereas, for the NaClO_4_/PC electrolyte, a severe capacity fading was observed after the first cycle and retained only 11.5% after 800 cycles. This might be due to large polarization on the MnO_2_ surface due to decomposition of PC.^46^ Although, it is reported that the EC+DMC-based electrolyte is reactive against Na and unsuitable for SIBs.^47^ The NaPF_6_/EC+DMC electrolyte shows better electrochemical performance in terms of excellent stability with a moderate capacity of MnO_2_ than the NaClO_4_/PC electrolyte. The stability reported by this study is much higher than the other literature reports of MnO_2_ for SIBs (Table S1).

Electrochemical impedance spectra (EIS) were investigated to gain insights into the reaction kinetics and the charge transfer. Figure 5c illustrates the EIS measurements of MnO_2_ for both the electrolytes. The well fitted equivalent circuit model for the same is as shown in Figure S3. The internal resistance (*R*_s_) corresponds to the intersection point on the *X* axis, whereas the charge transfer resistance (*R*_CT_) is denoted by the semicircular arc in the high frequency region.^48−51^ The constant phase element (CPE) and Warburg resistance (*W*) are associated with the double layer component inside pores and diffusion in the MnO_2_ nanostructure.^52,53^ The *R*_L_ component associates with lekage resistance during electrochemical activities. It is observed that the obtained value of *R*_CT_ for NaClO_4_/PC is 43.4 Ω (Figure 5c), which is smaller than that of NaPF_6_/EC+DMC (46.1 Ω) as presented in Table S2, suggesting slightly improved charge transfer speed and fast Na^+^ ions diffusion through the electrode-electrolyte interface. As shown in the Bode plot (Figure 5d), the MnO_2_ nanorods electrodes’ slope values at the high- (<1) and low-frequency (∼0) regions suggest that MnO_2_ has a faster sodium ion diffusion rate.^54,55^

### DFT Studies

**Figure 6:**
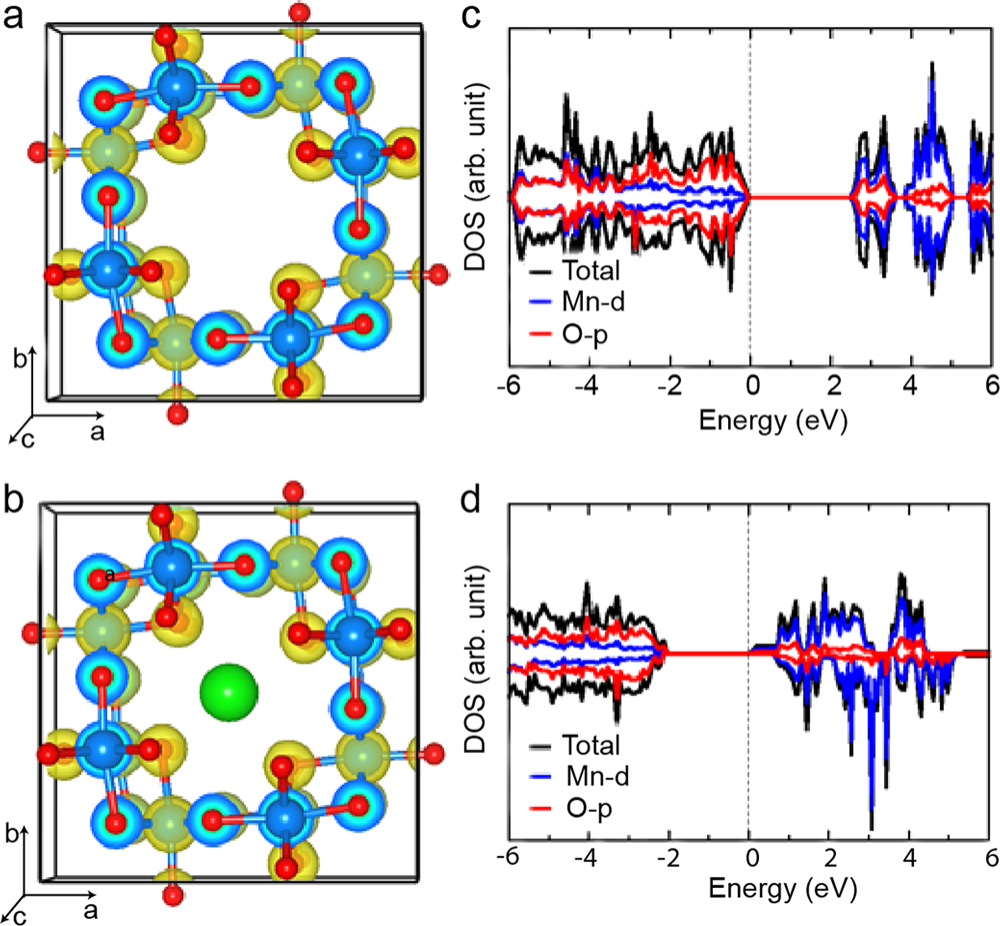
Charge density distribution for (a) pristine and (b) Na-intercalated α-MnO_2_. (c) Total (black line) and projected DOS on Mn ions (blue line) and O ions (red line) in pristine and (d) Na-intercalated α-MnO_2_.

Further insights into the electronic structure and Na ion diffusion in α-MnO_2_ were gained through first-principles DFT calculations. Both the pristine and Na ions intercalated α-MnO_2_ were modeled as a tetragonal crystal structure with an antiferromagnetic spin configuration, as shown in Figure 6a,b. A full unit cell relaxation yielded a strain-free α-MnO_2_ with lattice parameters *a* = *b* = 9.763 Å, *c* = 2.872 Å, in good agreement with the experimental data in the present and previous studies.^56,57^ The Na ion intercalation maintained the tetragonal structure with only slight contraction (1.53%) of the unit cell volume, the lattice parameters predicted at *a* = *b* = 9.715 Å and *c* = 2.856 Å. The predicted electronic band gap of pristine α-MnO_2_ is 2.42 eV (Figure 6c), and the experimental value of 2.23 eV is in good agreement.^58^ The valence band edge of MnO_2_ consists mainly of O *p* states, whereas Mn d states dominate the conduction band edge. Adding one Na atom puts one additional electron into the system, which caused a shift of the Fermi level near the conduction band (CB) edge and a reduction in the band gap to 2.01 eV. The charges on the O atoms located in the one-dimensional tunnel close to the Na ions have increased. Bader population analysis (Table S3) shows that the four oxygens closest to the Na ion received additional charges of 0.2 e^–^ each. The charge density in the pristine α-MnO_2_ and Na-intercalated α-MnO_2_ systems is shown in Figure 6a,b.

The voltage produced by the electrochemical process of Na + MnO_2_ → NaMnO_2_ was determined based on the following energy difference , where *E*(NaMnO_2_) is the energy of the α-MnO_2_ intercalated with a Na ion, *E*(MnO_2_) is the energy of the pristine α-MnO_2_, *E*(Na) is the energy a Na atom, and *N*_electrons_ is the number of electrons transferred with the cation.^59^ Volume and entropy effects are not included considering they are typically negligible (≤0.1 V).^60^ The reaction of Na with α-MnO_2_ produced a voltage of 3.42 V, similar to the earlier reported voltage of 3.23 V by Tompsett et al.^61^ The kinetics of Na diffusing in the α-MnO_2_ was evaluated performing a climbing-image nudge elastic band (CI-NEB) calculation, where seven images were constructed for interpolating the Na diffusion path along the 1-D tunnel within the 1 × 1 × 2 α-MnO_2_ as shown in Figure S4. The calculated migration energy barrier of Na was 0.21 eV, which is relatively low for practical battery applications.^62^ This is consistent with previous theoretical predictions by Tompsett et al., who reported the migration barriers for both Li ions and Na ions to be less than 0.3 eV.^61^

## Conclusions

In summary, α-MnO_2_ nanorods prepared using a hydrothermal method exhibited impressive electrochemical performance as a cathode material for SIBs when charged and discharged in the voltage range of 1–4 V, rendering an initial discharge capacity of 181 mAh·g^–1^ at C/20, however, with low capacity retention in NaClO_4_/PC electrolyte. In comparison, the cycling performance is improved (58.6% after 800 cycles) when using NaPF_6_/EC+DMC electrolyte, though it suffers from less capacity (109 mAh·g^–1^). Furthermore, the discharge capacities changed when cycled at different rates. Such superior electrochemical performance of the α-MnO_2_ material utilized in SIBs promises to solve stability issues. The α-MnO_2_ displays a high voltage of 3.42 V for Na intercalation and facile diffusion kinetics with a diffusion barrier as low as 0.21 eV as predicted from first-principles DFT calculations. The experimental results suggest that the hierarchically organized α-MnO_2_ sufficiently satisfies the stringent requirements of electrode materials in constructing high-power and long-term life batteries for energy storage applications. Further, we expect that novel efforts in finding newer electrolytes and their additives will overcome the present hurdles in achieving higher energy density with stable cycle performance for SIB.

## Experimental Section

### Synthesis of MnO_2_ Nanorods

The chemical oxidation of a manganese precursor (manganese(II) acetate) with ammonium persulfate in alkaline conditions leads to MnO_2_ synthesis. First, sodium hydroxide (100 mL) of desired concentration was prepared. This transparent solution of sodium hydroxide is taken in two separate beakers: one for ammonium persulfate precursor and the other for manganese precursor. After stirring the two solutions separately, the beaker containing ammonium persulfate solution is slowly poured into the manganese precursor solution. This solution is transferred into a Teflon-lined stainless-steel autoclave. This solution is kept at 180 °C for 14 h. The resultant precipitate was washed with ethanol and water and dried at 60 °C for 12 h. Finally, this product is annealed at 300 °C in the air atmosphere for 4 h. The chemical reactions involved in the formation of manganese dioxide are as per previous reports.^13^

### Material Characterization

An X-ray diffractometer (XRD) was used to identify the phase of the prepared MnO_2_ powder using the D8 Advance Bruker instrument using monochromatic Cu Kα radiation (λ = 1.5406 Å). X-ray Rietveld refinement was carried out with the FullProf program using the pseudo-Voigt profile function. Raman studies were performed with a LabRAM HR, 532 nm laser excitation. X-ray photoelectron spectroscopy (XPS) analysis was carried out for the samples, using an ESCALAB 250 (ThermoElectron, Al Kα) spectrometer. The electron microscopy analysis was carried out using a field emission scanning electron microscope (Hitachi S-4800) and a high-resolution transmission electron microscope (TECNAI G2 20 Twin, FEI). The details about electrochemical analysis and first-principles calculations are as per Note S2.

## Supporting Information Available

Details of XPS peak deconvolution, details of electrochemical and computational analysis, Raman spectrum, EDS elemental analysis, equivalent circuit for EIS spectra, energy profile of Na ion migration path through the α-MnO_2_, table of comparative performance for cycle life, equivalent circuit parameters, Bader charge analysis results of pristine and Na-intercalated α-MnO_2_ (PDF)
