# Supplementary material for: High Stability and Long Cycle Life of Rechargeable Sodium-Ion Battery Using Manganese Oxide Cathode: A Combined Density Functional Theory (DFT) and Experimental Study
Source: ACS Appl Mater Interfaces. 2021 Feb 25;13(9):11433–41. doi: 10.1021/acsami.0c21081 (PMC8023529; doi:10.1021/acsami.0c21081)
Supplement: Supplementary file 1 — am0c21081_si_001.pdf [file am0c21081_si_001.pdf]

Supporting Information

High stability and long cycle life of rechargeable sodium-ion battery using manganese oxide cathode: A combined density functional theory (DFT) and experimental study

Bidhan Pandit,[a,b]* Sachin R. Rondiya,[c] Nelson Y. Dzade,[c] Shoyebmohamad F. Shaikh,[d] Nitish Kumar,[e] Emad S. Goda,[f] Abdullah A. Al-Kahtani,[d] Rajaram S. Mane,[g] Sanjay Mathur,[h] Rahul R. Salunkhe[e,]*

[a]*Institut Charles Gerhardt Montpellier (ICGM), Université de Montpellier, Place Eugène Bataillon, Montpellier 34095, Cedex 5, France*

[b]*Department of Materials Science and Engineering and Chemical Engineering, Universidad Carlos III de Madrid, Avda. Universidad 30, E-28911 Leganés, Madrid, Spain*

[c]*School of Chemistry, Cardiff University, Main Building, Park Place, Cardiff, CF10 3AT, Wales, United Kingdom*

[d]*Department of Chemistry, College of Science, King Saud University, P.O. Box 2455, Riyadh 11451, Saudi Arabia*

[e]*Department of Physics, Indian Institute of Technology Jammu Jagti PO Nagrota, NH 44, Jammu 181221, J & K, India*

[f]*Fire Protection Laboratory, National Institute of Standards, 136, Giza 12211, Egypt*

[g]*Swami Ramanand Teerth Marathwada University, Nanded, 431606, M.S., India*

[h]*Chemistry Department, Institute of Inorganic Chemistry, University of Cologne, Greinstr. 6, 50939, Cologne, Germany*

Corresponding authors E-mail*

physics.bidhan@gmail.com (Dr. Bidhan Pandit)

rahul.salunkhe@iitjammu.ac.in (Dr. Rahul R. Salunkhe)

Note S1. Details of XPS peak deconvolution

The XPS peaks were deconvoluted using Voigt functions, which is a convolution of a Gaussian-Lorentzian peak shape. A manual subtraction of the background (Shirley or linear background as per requirement) is needed before the convolution. Here we considered Shirley's background for XPS deconvolution. A script was then formulated with the least-squares fitting procedure to determine the position, area, and width (FWHM) of the fitted peak cf the data. Even XPS core level spectra usually contain many peaks, fitting a set of different peaks, using as many constraints as possible. For instance, the FWHM of several other species can sometimes be approximated as equal. The area ratio of spin-orbit split peaks can be fixed (1:2 for p orbitals, 2:3 for d orbitals, 3:4 for f orbitals). FWHM of spin-orbit split peaks can also be fixed at a ratio as several chemical species of the doublets appeared with specific binding energy separation. These are all the details about the deconvolution and calibration process for fitting XPS peaks.

NOTE S2. DETAILS OF ELECTROCHEMICAL AND COMPUTATIONAL ANALYSIS

Electrochemical analysis

The electrochemical performance was examined in standard coin cells assembled in a glove box. Electrode formulation was done using a mixture of carbon black and vapor grew carbon fibers (VGCF-H) conductive additives, and Poly(vinylidene fluoride) (PVDF) (Solef 5130, Solvay) binder, in 1-Methyl-2-pyrrolidinone (NMP, anhydrous, 99.5% Sigma-Aldrich). A planetary ball-milling homogeneously mixed a slurry containing the active material (70%), the binder (12%), and the conductive additive (18%) for 1 h, tape cast on a 150 mm thick aluminum foil, dried at room temperature, and finally at 120 °C under vacuum overnight. The electrode's active material mass loading varied from 1.8-3.4 mg·cm^{-2}, depending on the percentage of active material in the formulation. The electrochemical tests vs. Na were performed against a counter-electrode of pure sodium (from Sigma-Aldrich), using 1 M $NaPF_6$ in ethylene carbonate and dimethyl carbonate (EC:DMC) in 5% Fluoro Ethylene Carbonate (FEC) and $NaClO_4$ in PC with 5% FEC as electrolytes. Whatman glass-fiber was used as a separator. All tests were carried out using a multichannel VMP system under galvanostatic mode in the operation window from 1.5 to 4.0 V vs. Na^+/Na at nC rate (*i.e.*, n = 1, 1 Na in one hour) at room temperature.

First-principles calculations

The Vienna Ab initio Simulation Package (VASP) was used to perform the spin-polarized DFT calculations.[1,2] The interactions between the ionic core and the valence electrons were described with the projected augmented wave (PAW) method.[3] The Perdew–Burke–Ernzerhof (PBE) generalized gradient approximation (GGA) functional was used to calculate the electronic exchange-correlation potential.[4] An energy cutoff of 600 eV and Monkhorst-Pack[5] *k*-point mesh of 7 × 7× 3 was used to sample the Brillouin zone of α-MnO_2. Geometry optimizations were performed based on the conjugate-gradient algorithm until the residual Hellmann–Feynman forces on all relaxed atoms reached 10^{-3} eV Å^{-1}. To precisely reproduce the experimentally known density of state (DOS) and band gaps features of α-MnO_2, the screened hybrid functional HSE06[6] was used with the exchange value of 25%. The tetrahedron method with Bloch correction was used to calculate the projected density of states (PDOS).[7]

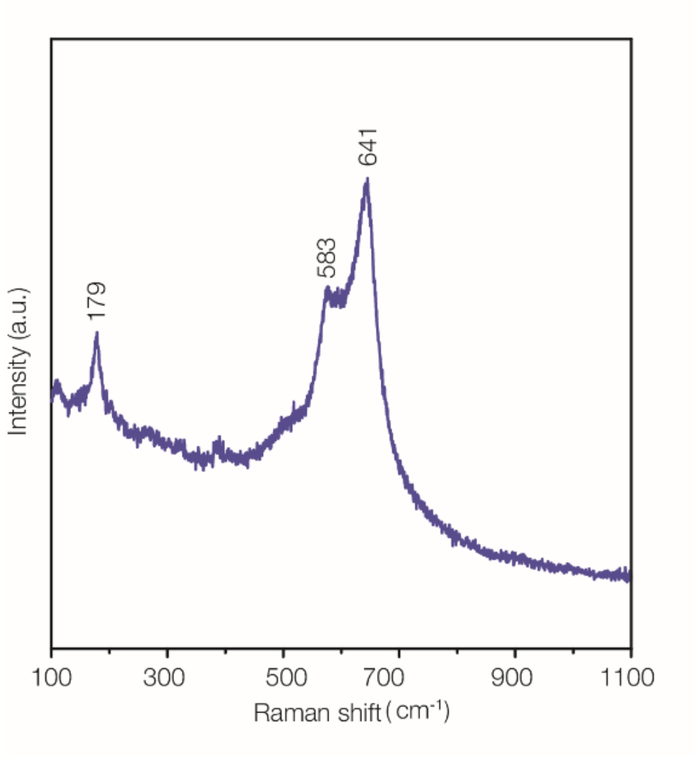

Figure S1. Raman spectrum of MnO_2 nanorods.

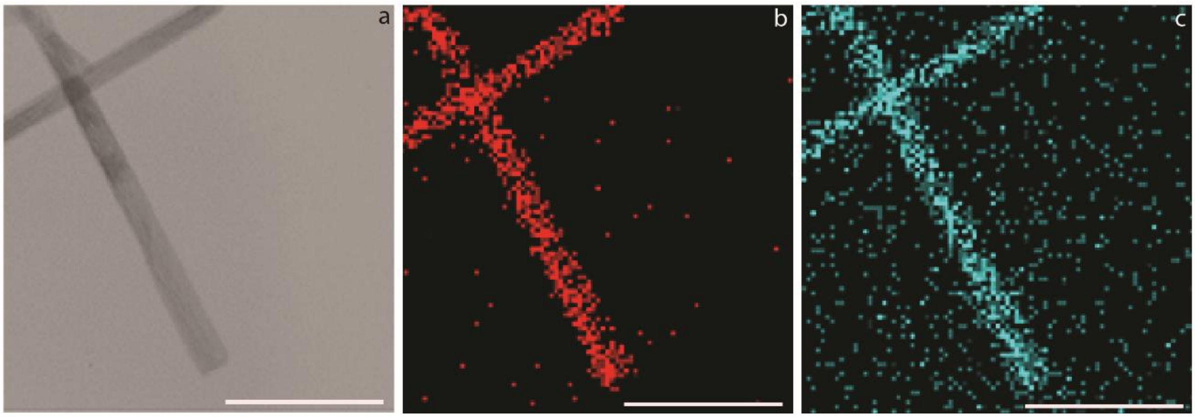

Figure S2. EDS elemental analysis of MnO_2 nanorods. (a). Actual TEM Image of the sample. (b) Red color indicates Mn content in the sample.(c) The blue color indicates oxygen content in the sample. The scale bar is 125 nm in length.

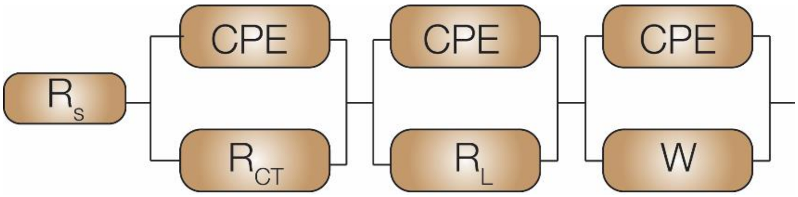

Figure S3. Equivalent circuit model for the fitted EIS spectra in Figure 5c in the main text.

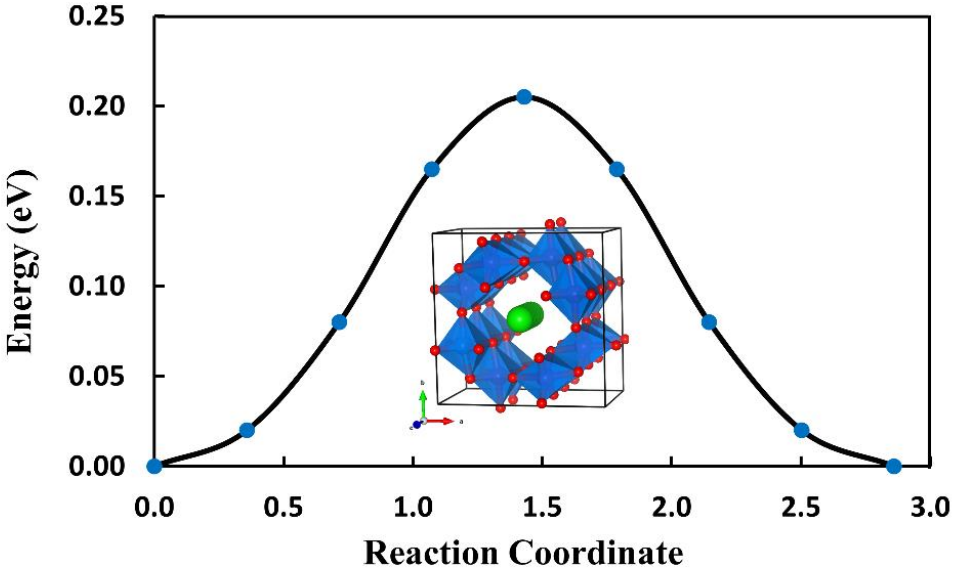

Figure S4. Energy profile of Na ion migration path through the α-MnO_2 1×1×2 supercell. The insert shows the Na ion (green atoms) position through the C1-NEB path.

Table S1 Comparative performance of MnO_2 based electrodes for sodium-ion battery applications

Material	Method	Electrolyte	Voltage window	Capacity ($mAh \cdot g^{-1}$)		Stability		Ref.
				Capacity ($mAh \cdot g^{-1}$)	Rate ($mA \cdot g^{-1}$)	Retention (%)	cycle	
δ-MnO_2	Chemical	1M $NaClO_4$/EC + DMC (1:1)	0-3	334.3	50	98	500	[8]
α-MnO_2	Microwave-assisted	1M $NaPF_6$/EC:DEC (1:1)	1.3-3.8	247	5	54	50	[9]
β-MnO_2	Hydrothermal	1M $NaClO_4$/EC + PC (1:1)	1-4.3	350	20	54.9	100	[10]
α-MnO_2	Hydrothermal	1M $NaClO_4$/EC:PC (1:1)	1-4.3	278	20	27	100	[11]
β-MnO_2	Hydrothermal	1M $NaClO_4$/EC:PC (1:1)	1-4.3	298	20	48.7	100	
MnO_2	Hydrothermal	1M $NaClO_4$/EC:DEC (1:1)	0-3	487.8	50	36.3	100	[12]
MnO_2	Hydrothermal	1M $NaClO_4$/EC:PC (1:1)	0-3	760.8	50	23	400	[13]
MnO_2	Wet chemical	1M $NaClO_4$/EC:DMC (1:1)	1.5-4	132.2	0.1 C	111	100	[14]
α-MnO_2	Hydrothermal	1M $NaPF_6$/EC:DMC (1:1) + FEC (5%)	1-4	109	0.05 C	58.6	800	Present work
α-MnO_2	Hydrothermal	1M $NaClO_4$/PC + FEC (5%)	1-4	181	0.05 C	11.5	800	

Table S2 Equivalent circuit parameters obtained from fitted EIS spectra.

Materials	Electrolyte	R_S	R_{CT}
α-MnO_2	$NaPF_6$/EC + DMC + FEC (5 %)	18.8	46.1
	$NaClO_4$/PC + FEC (5 %)	18.8	43.4

Table S3 Bader charge analysis results of pristine and Na intercalated α-MnO_2. The four oxygen atoms closest to the Na ion in the 1D channel are highlighted in the last four rows.

**Atoms**	***q*** **(MnO_2)**	***q*** **(Na-MnO_2)**	**Δ*q***
Mn1	2.0073	1.9974	0.0099
Mn2	2.0073	1.9952	0.0121
Mn3	2.0073	2.0175	-0.0102
Mn4	2.0073	2.0075	-0.0002
Mn5	2.0073	2.0165	-0.0092
Mn6	2.0073	2.0285	-0.0212
Mn7	2.0073	1.9967	0.0106
Mn8	2.0073	1.9974	0.0099
O1	-1.0655	-1.0584	-0.0071
O2	-1.0661	-1.0558	-0.0103
O3	-1.0662	-1.0499	-0.0163
O4	-1.0655	-1.0562	-0.0093
O5	-1.0661	-1.0965	0.0304
O6	-1.0655	-1.0948	0.0293
O7	-1.0662	-1.0853	0.0191
O8	-1.0655	-1.088	0.0225
O9	-0.9419	-0.9647	0.0228
O10	-0.9419	-0.9709	0.029
O11	-0.9409	-0.9906	0.0497
O12	-0.9409	-0.9839	0.043
O13	-0.9409	-1.1384	0.1975
O14	-0.9409	-1.1356	0.1947
O15	-0.9419	-1.1467	0.2048
O16	-0.9419	-1.137	0.1951

References

(1) Kresse, G.; Hafner, J. Ab Initio Molecular Dynamics for Liquid Metals. *Phys. Rev. B* **1993**, *47*, 558–561.

(2) Kresse, G.; Joubert, D. From Ultrasoft Pseudopotentials to the Projector Augmented-Wave Method. *Phys. Rev. B* **1999**, *59*, 1758–1775.

(3) Blöchl, P. E. Projector Augmented-Wave Method. *Phys. Rev. B* **1994**, *50*, 17953–17979.

(4) Perdew, J. P.; Burke, K.; Ernzerhof, M. Generalized Gradient Approximation Made Simple. *Phys. Rev. Lett.* **1997**, *78*, 1396–1396.

(5) Monkhorst, H. J.; Pack, J. D. Special Points for Brillouin-Zone Integrations. *Phys. Rev. B* **1976**, *13*, 5188–5192.

(6) Krukau, A. V.; Vydrov, O. A.; Izmaylov, A. F.; Scuseria, G. E. Influence of the Exchange Screening Parameter on the Performance of Screened Hybrid Functionals. *J. Chem. Phys.* **2006**, *125*, 224106.

(7) Blöchl, P. E.; Jepsen, O.; Andersen, O. K. Improved Tetrahedron Method for Brillouin-Zone Integrations. *Phys. Rev. B* **1994**, *49*, 16223–16233.

(8) Liu, F.; Xiao, Y.; Liu, Y.; Han, P.; Qin, G. Mesoporous MnO_2 Based Composite Electrode for Efficient Alkali-Metal-Ion Storage. *Chem. Eng. J.* **2020**, *380*, 122487.

(9) Huang, J.; Poyraz, A. S.; Lee, S. Y.; Wu, L.; Zhu, Y.; Marschilok, A. C.; Takeuchi, K. J.; Takeuchi, E. S. Silver-Containing α-MnO_2 Nanorods: Electrochemistry in Na-Based Battery Systems. *ACS Applied Materials and Interfaces* **2017**, *9*, 4333–4342.

(10) Su, D.; Ahn, H.-J.; Wang, G. β-MnO_2 Nanorods with Exposed Tunnel Structures as High-Performance Cathode Materials for Sodium-Ion Batteries. *NPG Asia Mater.* **2013**, *5*, e70–e70.

(11) Su, D.; Ahn, H. J.; Wang, G. Hydrothermal Synthesis of α-MnO_2 and β-MnO_2 Nanorods as High Capacity Cathode Materials for Sodium Ion Batteries. *J. Mater. Chem. A* **2013**, *1*, 4845–4850.

(12) Zhang, Z.; Zhao, X.; Li, J. Facile Synthesis of Nanostructured MnO_2 as Anode Materials for Sodium-Ion Batteries. *ChemNanoMat* **2016**, *2*, 196–200.

(13) Li, H.; Liu, A.; Zhao, S.; Guo, Z.; Wang, N.; Ma, T. In Situ Growth of a Feather-like MnO_2 Nanostructure on Carbon Paper for High-Performance Rechargeable Sodium-Ion Batteries. *ChemElectroChem* **2018**, *5*, 3266–3272.

(14) Zhou, Y.; Chen, T.; Zhang, J.; Liu, Y.; Ren, P. Amorphous MnO_2 as Cathode Material for Sodium-Ion Batteries. *Chinese J. Chem.* **2017**, *35*, 1294–1298.
